# Calculating and estimating second cancer risk from breast radiotherapy using Monte Carlo code with internal body scatter for each out‐of‐field organ

**DOI:** 10.1002/acm2.13060

**Published:** 2020-10-30

**Authors:** Takeshi Takata, Kenshiro Shiraishi, Shinobu Kumagai, Norikazu Arai, Takenori Kobayashi, Hiroshi Oba, Takahide Okamoto, Jun’ichi Kotoku

**Affiliations:** ^1^ Graduate School of Medical Care and Technology Teikyo University 2‐11‐1 Kaga, Itabashi‐ku Tokyo 173‐8605 Japan; ^2^ Department of Radiology Teikyo University School of Medicine 2‐11‐1 Kaga, Itabashi‐ku Tokyo 173‐8605 Japan; ^3^ Central Radiology Division Teikyo University Hospital 2‐11‐1 Kaga, Itabashi‐ku Tokyo 173‐8605 Japan

**Keywords:** breast cancer, low‐dose bath, Monte Carlo simulation, radiotherapy, second cancer risk

## Abstract

Out‐of‐field organs are not commonly designated as dose calculation targets during radiation therapy treatment planning, but they might entail risks of second cancer. Risk components include specific internal body scatter, which is a dominant source of out‐of‐field doses, and head leakage, which can be reduced by external shielding. Our simulation study quantifies out‐of‐field organ doses and estimates second cancer risks attributable to internal body scatter in whole‐breast radiotherapy (WBRT) with or without additional regional nodal radiotherapy (RNRT), respectively, for right and left breast cancer using Monte Carlo code PHITS. Simulations were conducted using a complete whole‐body female model. Second cancer risk was estimated using the calculated doses with a concept of excess absolute risk. Simulation results revealed marked differences between WBRT alone and WBRT plus RNRT in out‐of‐field organ doses. The ratios of mean doses between them were as large as 3.5–8.0 for the head and neck region and about 1.5–6.6 for the lower abdominal region. Potentially, most out‐of‐field organs had excess absolute risks of less than 1 per 10,000 persons‐year. Our study surveyed the respective contributions of internal body scatter to out‐of‐field organ doses and second cancer risks in breast radiotherapy on this intact female model.

## 1 Introduction

Breast‐conserving therapy requiring radiation therapy is the standard of care for early‐stage breast cancer. Whole‐breast radiotherapy (WBRT) and regional nodal radiotherapy (RNRT) are first‐line treatments. Nevertheless, despite their benefits, doses attributed to head scatter, collimator transmission, and internal body scattering can cause second cancer and deterministic effects in peripheral organs. Reports of experiment cohorts have described a significant association between radiotherapy for the breast and second cancers in organs adjacent to the breast, that is, the lung, esophagus, and thyroid.^1^, ^2^, ^3^ However, an association between radiation therapy and second cancer risk in out‐of‐field organs, remains unclear. Components of out‐of‐field doses are internal body scattering, head leakage, collimator transmission, and room scattering. Some reports of the literature have described that internal body scattering is a dominant component of out‐of‐field doses.^4^, ^5^ It is reported that internal body scattering contributes 70% of the out‐of‐field dose for conventional radiotherapy techniques.^5^ Contributions of head leakage and collimator transmission radiation are described in the literature.^5^, ^6^, ^7^ Earlier findings suggest that the magnitude of leakage head scatter that contributes to the out‐of‐field dose can be reduced by placing a lead shield over the critical area.^8^, ^9^ Internal body scatters, however, cannot be shielded. Knowledge of exposure attributable to internal body scatter is crucially important for radiation risk management. Nevertheless, measuring the dose directly in each organ in a patient body is impossible.

Out‐of‐field organ doses must be managed to mitigate second cancer risk, just as they are for in‐field organs, but they are not calculated in general radiotherapy treatment planning. Moreover, treatment planning system (TPS) accuracy is usually limited to a few centimeters outside the field edge.^10^, ^11^ Earlier studies have estimated out‐of‐field absorbed doses using anthropomorphic phantom measurements or TPS calculations on patient computed tomography (CT) images. Kry et al. calculated doses for out‐of‐field organs and estimated second cancer risks after prostate radiotherapy using TPS.^12^ Howell et al. evaluated the accuracy of TPS for out‐of‐field dose calculation compared to measurements using an anthropomorphic phantom.^10^ Zeverino et al. summarized the peripheral organ dose in breast radiotherapy depending on the planning technique for several TPSs.^13^ Kourinou et al. measured the contribution of internal body scatter to the fetal dose using an anthropomorphic phantom.^14^ These experimental reports have described approximate doses in out‐of‐field organs in TPS for breast cancer, but out‐of‐field organ doses have not been established based on physical simulation with a complete whole‐body model.

Some refined analytical models and systems are able to predict out‐of‐field doses.^15^, ^16^ However, scattering in the complex structure of the human body is not considered. Also, the dose of a specific organ is not a calculation target.

Monte Carlo (MC) simulation, which is well known as a physically accurate dose calculation method, has been used in some earlier studies to calculate out‐of‐field absorbed doses. Reda et al. used a simple anthropomorphic phantom to calculate the doses for some organs for one situation of breast radiotherapy.^17^ Joosten et al. calculated out‐of‐field organ absorbed doses by simulating irradiation of left breast radiotherapy on a CT‐based female phantom that did not include forearms or lower limbs.^18^, ^19^ Abo‐Madyan et al. estimated the second cancer risk for the breast and lung after breast radiotherapy.^20^ However, no report of the relevant literature describes a study validating irradiation effects for regional lymph nodes from a low‐dose bath or a study estimating the second cancer risk for out‐of‐field organs mainly attributed to internal body scatter in radiotherapy for the breast in a complete whole‐body MC simulation.

This study specifically undertook simulation of all organ absorbed doses by internal body scatter from conventional radiotherapy techniques using WBRT alone and WBRT plus RNRT, with calculation of second cancer risks in these radiotherapies using the concept of the organ equivalent dose (OED) ^21^ with Japanese atomic bomb survivor data. The radiation dose was calculated for a complete whole‐body voxel model for Japanese female adults ^22^ using the MC code Particle and Heavy Ion Transport code System (PHITS, ver. 3.02).^23^

## 2 Methods

### 2.A Whole‐body female model

**Fig. 1:**
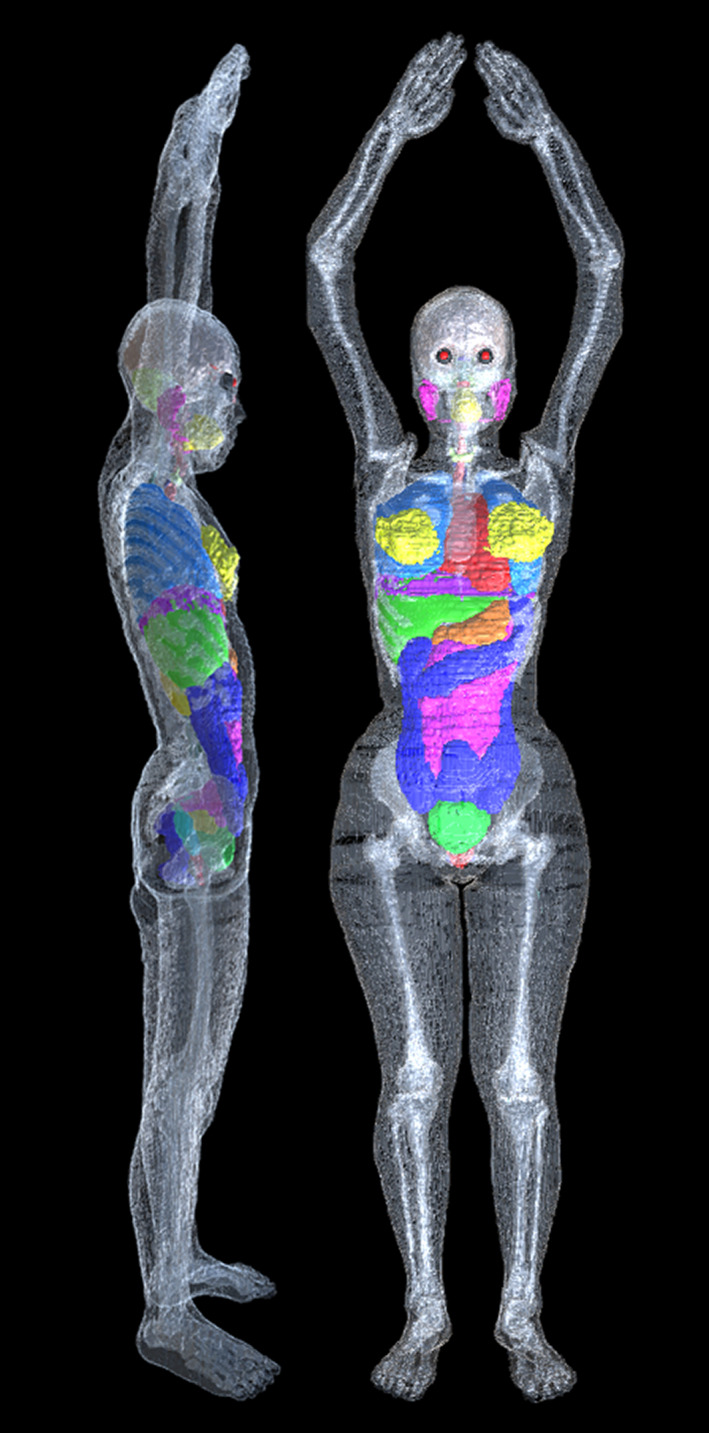
Whole‐body female model designed by the National Institute of Information and Communications Technology.^22^ Fat, muscle, blood, and skull are not visible in this figure..

Simulation of the particular organ dose distributions in a female body requires a full standard whole‐body model. For this study, we used the whole‐body voxel model for a Japanese female adult designed in 2003 by the National Institute of Information and Communications Technology.^22^, ^24^ This model was constructed based on magnetic resonance images of a female Japanese person. The model height and weight approximate the average body size for a Japanese female adult (159.1 cm and 52.6 kg). The model, which consists of 2 × 2 × 2 mm^3^ (coronal, axial, sagittal) voxels, is segmented into 47 tissues and organs including the contents of digestive organs. Free‐form deformation software ^24^ for postural modification of the model is combined with the whole‐body model package. The model posture was modified for dose calculation: it was set to resemble a realistic position of raising both arms above the head (Fig. 1). We assigned mass density and compositions for all tissues and organs based on the International Commission on Radiological Protection (ICRP) publication 110^25^ and allocated air around the model.

### 2.B Treatment planning

The WBRT plans consisted of two opposing tangential fields with two additional opposing subfields for the breast using multileaf collimators (MLCs), that is, “field‐in‐field technique.” RNRT plans had two opposing regional lymph node fields using MLCs. The prescribed dose of WBRT is 50 Gy to the breast in 25 fractions. That of RNRT is 50 Gy to regional (subclavicular and internal mammary) lymph nodes in 25 fractions. For treatment planning, a combination of 4‐ and 6‐MV x‐ray beams was used. Above is a standard of care for breast cancer patients at our institution. The convolution–superposition algorithm, a model‐based algorithm, was used for dose calculation in TPS. The calculation grid size was equal to the voxel model (2 × 2 × 2 mm^3^). Many internal organs are asymmetrically shaped and positioned relative to the left and right sides of the body. Asymmetry might be a cause of different organ doses found for irradiation of the left and right sides. To evaluate the difference, WBRT alone and WBRT plus RNRT treatment plans were generated, respectively, for the left and right sides using software (Pinnacle^3^ ver. 14.0; Philips Medical Systems) TPS. A certified medical physicist generated these treatment plans for breast cancer for the whole‐body female model.

Conversion from a voxel format to DICOM format is necessary to plan treatments for the whole‐body voxel model on the TPS. For this study, voxel values in DICOM files were inversely assigned mass density of each tissue and organ from the CT‐density table. The outside region of the phantom was set to −1000 Hounsfield units (HU) as air.

### 2.C Monte Carlo dose calculation using PHITS

For this study, we chose a full MC simulation package that supports Message Passing Interface (MPI) protocols: PHITS ver. 3.02.^23^ PHITS incorporates Electron Gamma Shower Version 5 (EGS5), in which photon and electron transport were established as valuable tools to simulate a medical linac.^26^, ^27^ As incident x‐ray spectra, we used MC modeled photon fluence spectra reported by Shiekh‐Bagheri and Rogers.^28^ Beam information such as the leaf position, jaw position, and beam angle were obtained from DICOM RT files planned in Pinnacle^3^ TPS for the MC simulation. The whole‐body voxel model was downsampled to voxel size 2 × 2 × 4 mm^3^ (coronal, axial, sagittal) to input the model into PHITS and to reduce statistical uncertainty. To simulate only the dose contributed from internal body scatter, which is the principal component of out‐of‐field doses,^4^, ^5^ scattered radiation produced by the linear accelerator, for example, head scatter, collimator transmission, and MLC transmission, was not modeled for dose estimation in this study. To validate the beam source models, we compared the percentage depth dose (PDD) and beam profiles of 4‐ and 6‐MV x‐ray beams in water (50 × 50 × 50 cm^3^) for 10 × 10 cm^2^ field and 100 cm source‐to‐surface distance between the PHITS simulation and TPS.

The photon and electron cutoff energies were, respectively, 10 keV and 100 keV. The photon mean free path at 10 keV in liquid water is approximately 1.9 mm.^29^ The electron attenuation length at 100 keV in liquid water is approximately 0.14 mm.^30^ They were smaller than the voxel size.

Two billion incident photons for each simulated beam were used to achieve statistical uncertainties of less than 2% at the target breast conclusively. Generally, MC simulation requires a long duration and tremendous computational power to reduce statistical uncertainty. We accelerated simulation by parallel computing using a supercomputing system (Reedbush‐H, two 18 core processors, Xeon E5‐2695v4; Intel Corp, with 256 GB random access memory; Silicon Graphics International Corp.) with 72 MPI processes at the Information Technology Center of The University of Tokyo.

The simulated relative dose distributions by PHITS were rescaled to the prescribed dose (50 Gy) at each beam specification point. Our software developed in‐house extracted the specific organ dose distributions from the PHITS output files. The local gamma passing rate with a 3%/3 mm gamma criterion was calculated with the calculated organ‐dose distributions by PHITS and TPS.^31^

### 2.D Calculation of second cancer risk

We estimated the risk of second cancer by the excess absolute risk (EAR) optimized against the epidemiological data. The cancer incidence is assumed to be directly proportional to OED, according to the linear no‐threshold model. Therefore, EAR after radiotherapy is calculated as.

(1) $$EAR_{T}=EAR_{T_{0}}\cdot OED_{T}$$

where $EAR_{T}$ stands for EAR after radiotherapy for organ *T* (per 10,000 person‐year (PY)), $EAR_{T_{0}}$ denotes epidemiological EAR, and $OED_{T}$ signifies OED for organ *T*.^20^ EAR derived from Japanese atomic bomb survivor data for organ *T* (per 10,000 PY at 1 Gy at the age of 70 yr, following exposure at the age of 30 and 50 yr), as described by Preston et al.^32^

The OEDs for most out‐of‐field organs were calculated using the linear dose–response model mainly because the model accomplished good precision under 2 Gy.^33^ Most out‐of‐field organs in radiotherapy were exposed to less than 2 Gy in this study. Therefore, we calculated OED for the linear dose–response model as shown below.

(2) $$OED_{T,\text{linear}}=\frac{1}{V_{T}}\sum_{i=0}^{n}V_{D_{i}}\cdot D_{i}$$

That equation shows that, for each dose level, the summation from *i* equals zero to *n* of dose *D* in organ *T*, which has volume $V_{T}$. Also, $V_{D_{i}}$ is the volume corresponding to dose $D_{i}$.^33^

The mean absorbed doses for the target breast, the ipsilateral lung, the thyroid, the skin, the large intestine, the liver, the esophagus, and the spleen were sometimes found to be over 2 Gy in our study. For doses greater than 2 Gy, the dose–response relation for cancer is no longer linear. This model would generally overestimate the OED.^34^ It has often been hypothesized that the linear dose–response model is no longer applicable because of cell death at higher doses. The linear‐exponential dose–response model is proposed as one model to describe the dose–response at higher doses, as explained below.^21^, ^33^

(3) $$\begin{array}{c} OED_{T,\text{linear}‐\exp}=\frac{1}{V_{T}}\sum_{i=0}^{n}\left\{V_{D_{i}}\cdot D_{i}\cdot e^{‐\alpha D_{i}}\right\};\\ \alpha =0.044{\;\text{Gy}}^{‐1} \end{array}$$

Therein, model parameter α was estimated from a combined fit to the Japanese atomic bomb and Hodgkin cohorts.^21^, ^34^

## 3 Results

**Fig. 2:**
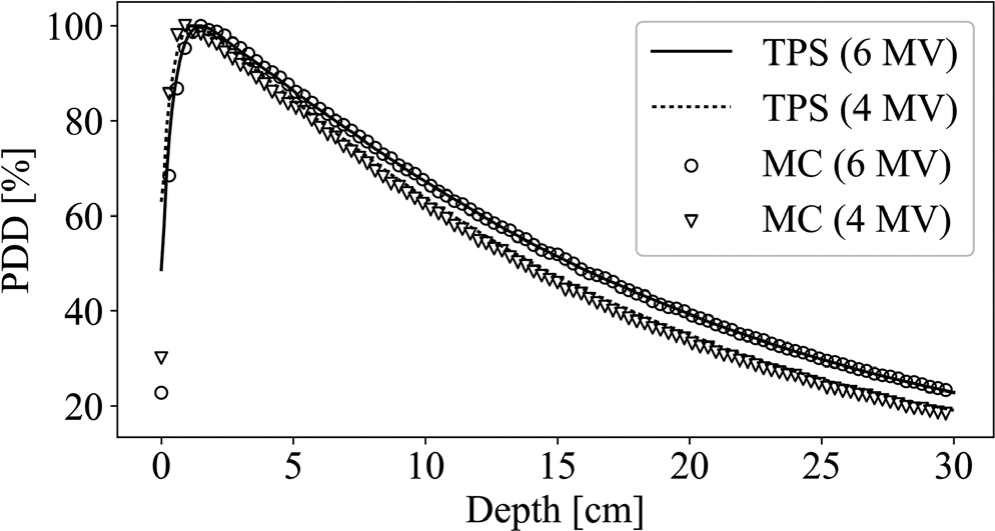
PDD comparison of the PHITS simulation and TPS calculation for 10 × 10 cm^2^ field

**Fig. 3:**
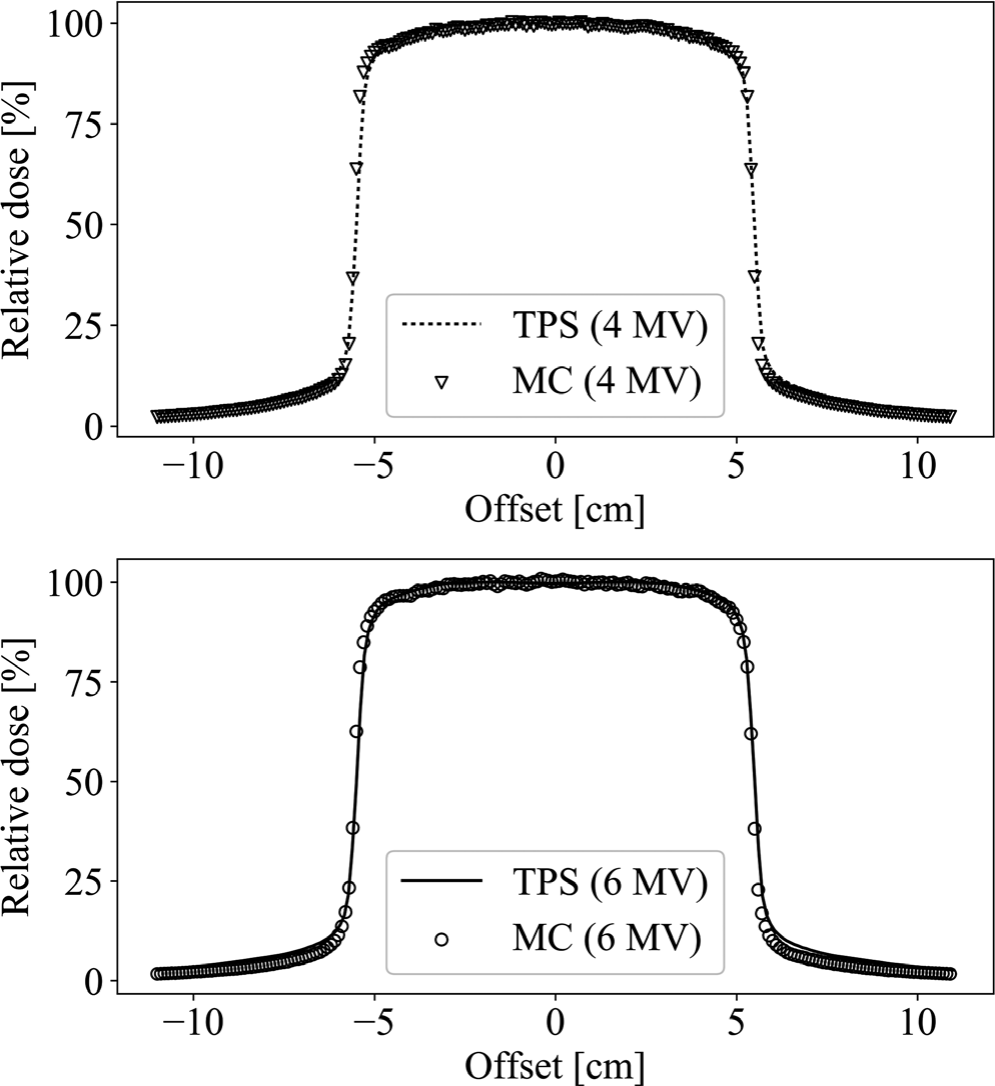
Beam profile comparison of the PHITS simulation and TPS calculation for 10 × 10 cm^2^ field at 10 cm depth.

Figure 2 and Fig. 3 respectively show comparisons of PDD and beam profiles at 10 cm depth between the PHITS simulation and TPS for beams used in this study. The PDD comparisons showed that PDD curves of PHITS and TPS matched well. However, a few differences were apparent around the surfaces of both curves. The PHITS beam profiles agreed well with those calculated using TPS in the central region and penumbrae. The PHITS beam edge values were very close to those of TPS.

Simulation with two billion incident photons took 31 h for each beam in field‐in‐field technique for the breast and 24 h for each beam for regional lymph node fields, on average. Each simulation for WBRT alone took about 124 h; each WBRT plus RNRT simulation took about 172 h on the Reedbush‐H supercomputing system.

Table 1 presents the simulated mean absorbed doses over the specified volume for the target breast from WBRT alone and WBRT plus RNRT using PHITS and TPS. The difference in the mean absorbed dose for the target breast was less than 1% between PHITS and TPS (convolution–superposition algorithm) in all treatment plans.

**Table 1:** Mean absorbed doses and the 68% confidence interval (CI) for the target breast from WBRT alone and WBRT plus RNRT for full MC PHITS and TPS.

		Mean dose for target breast			Gamma passing rate (3%/3 mm) [%]
		MC [Gy]	TPS [Gy]	Δ [%]	
Left side irradiation	WBRT	52.100 ± 0.594	51.666	−0.833	100
	WBRT plus RNRT	51.275 ± 0.728	51.579	0.593	100
Right side irradiation	WBRT	51.258 ± 0.569	51.462	0.398	100
	WBRT plus RNRT	51.101 ± 0.572	51.589	0.956	99.8

Difference Δ is given by the equation [Δ = (PHITS ‐ TPS)/ TPS × 100]. Gamma passing rates were calculated between PHITS and TPS.

**Fig. 4:**
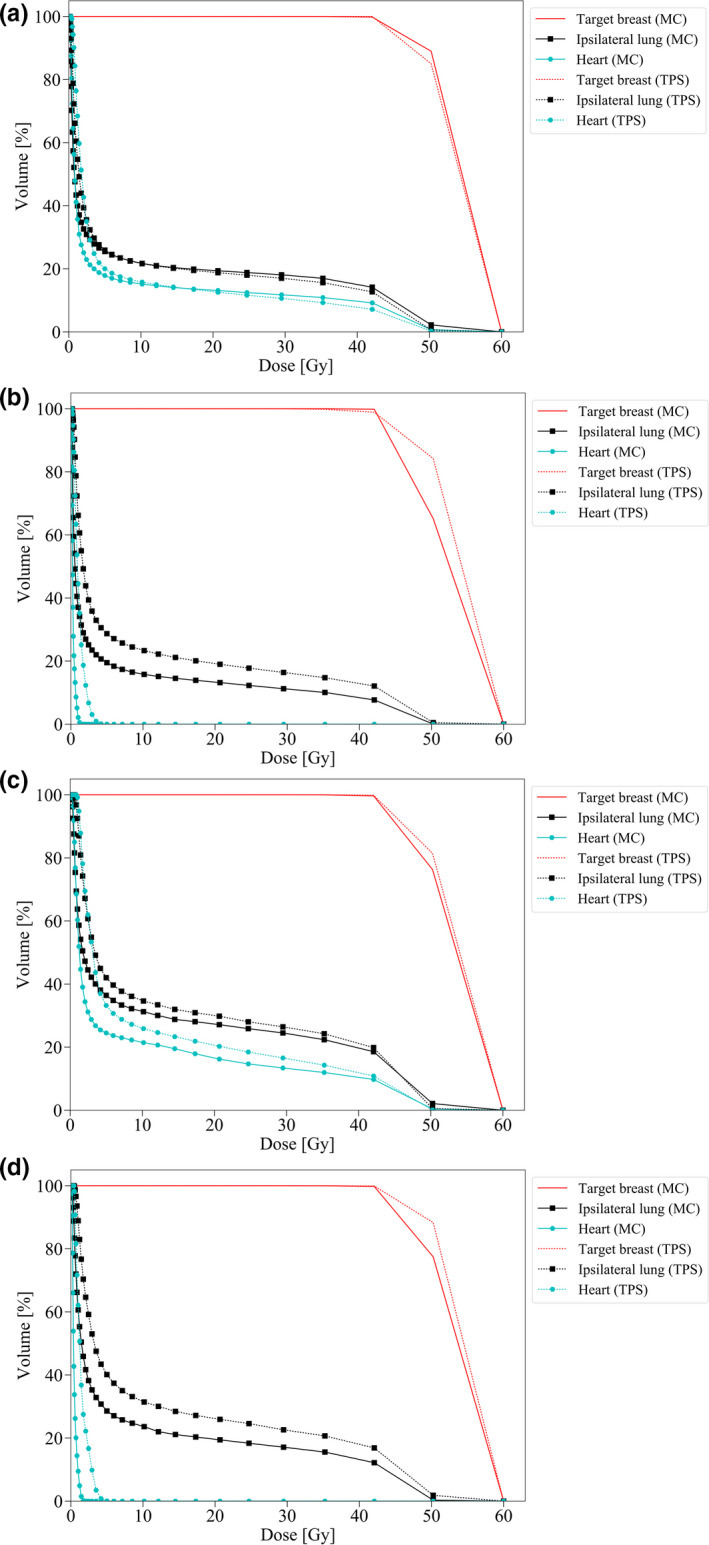
DVHs in the target breast and adjacent organs for full MC simulation PHITS and the convolution–superposition algorithm in TPS undergoing (a) WBRT for the left breast, (b) the right breast, (c) WBRT plus RNRT for the left breast, and (d) the right breast.

**Fig. 5:**
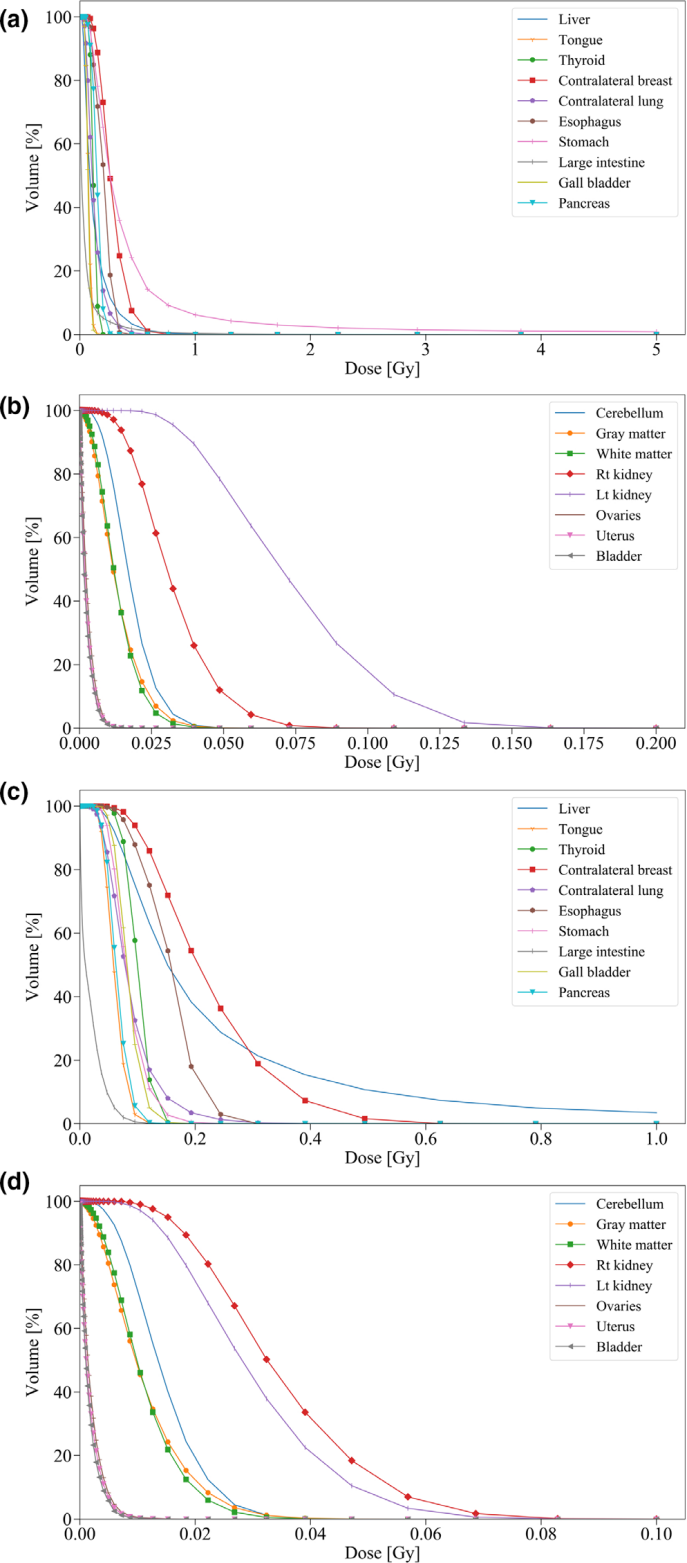
Part of simulated DVHs in out‐of‐field organs undergoing (a, b) WBRT for the left breast and (c, d) right breast.

**Fig. 6:**
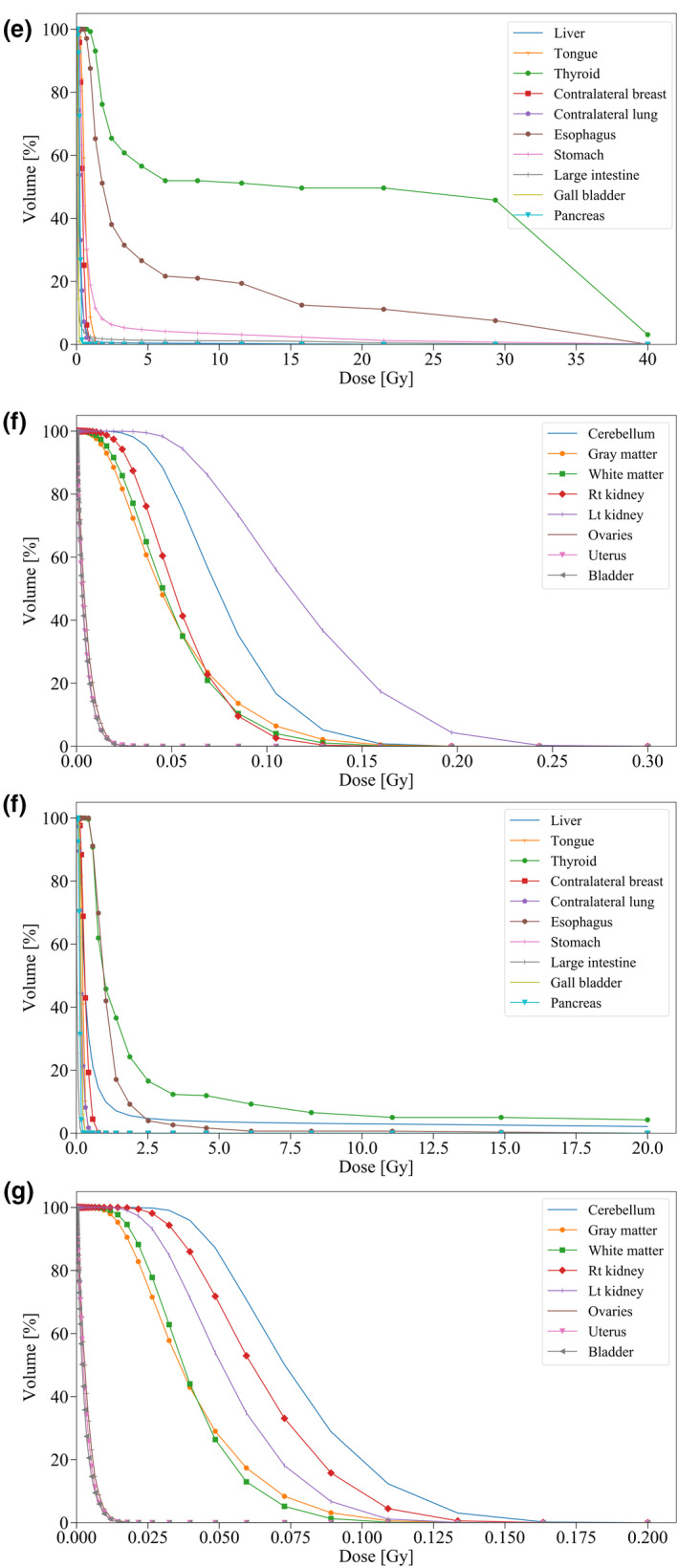
Continuance of Fig. 5. Part of simulated DVHs in out‐of‐field organs undergoing (e, f) WBRT plus RNRT for the left breast and (g, h) right breast.

The dose volume histograms (DVHs) for the target breast and organs near the radiation field by PHITS and TPS are shown in Fig. 4. The gamma index passing rate for them is shown in Table 1. The simulated dose for the target breast accorded well with TPS. Earlier reports of experimental studies have described that the convolution–superposition algorithm in TPS apparently overestimates the absorbed dose for the out‐of‐field organs around the chest.^35^, ^36^, ^37^ Figure 5 and Fig. 6 present part of DVHs for out‐of‐field organs simulated using PHITS (Simulated absorbed doses presented in Table 2). Full MC simulation was useful to survey the absorbed dose distribution from head to toe, although distant organs such as the genital area are outside the calculation area in TPS for radiotherapy for breast cancer.

**Table 2:** Simulated maximum and mean absorbed doses and the 68% CI for 43 organs undergoing WBRT alone and WBRT plus RNRT

	Left side irradiation				Right side irradiation				
	WBRT		WBRT plus RNRT		WBRT		WBRT plus RNRT		
	max [Gy]	mean [Gy]	max [Gy]	mean [Gy]	max [Gy]	mean [Gy]	max [Gy]	mean [Gy]	
Cerebellum	0.059 ± 0.014	0.018 ± 0.006	0.209 ± 0.041	0.077 ± 0.020	0.049 ± 0.011	0.015 ± 0.005	0.318 ± 0.054	0.113 ± 0.021	
Cornea	0.104 ± 0.021	0.041 ± 0.011	0.238 ± 0.051	0.142 ± 0.038	0.087 ± 0.017	0.032 ± 0.009	0.319 ± 0.055	0.153 ± 0.030	
Sclera	0.081 ± 0.017	0.031 ± 0.009	0.301 ± 0.074	0.133 ± 0.036	0.066 ± 0.016	0.026 ± 0.007	0.304 ± 0.053	0.119 ± 0.023	
Gray matter	0.060 ± 0.013	0.013 ± 0.005	0.265 ± 0.059	0.051 ± 0.017	0.056 ± 0.012	0.011 ± 0.004	0.293 ± 0.045	0.065 ± 0.016	
White matter	0.054 ± 0.018	0.013 ± 0.005	0.292 ± 0.071	0.050 ± 0.016	0.051 ± 0.012	0.011 ± 0.004	0.242 ± 0.034	0.065 ± 0.015	
Hypothalamus	0.039 ± 0.011	0.019 ± 0.006	0.112 ± 0.036	0.074 ± 0.021	0.026 ± 0.008	0.013 ± 0.004	0.160 ± 0.038	0.083 ± 0.017	
Eye lens	0.044 ± 0.011	0.032 ± 0.009	0.240 ± 0.065	0.155 ± 0.040	0.042 ± 0.011	0.025 ± 0.008	0.238 ± 0.049	0.133 ± 0.027	
Pineal gland	0.024 ± 0.009	0.016 ± 0.006	0.085 ± 0.021	0.057 ± 0.016	0.007 ± 0.003	0.006 ± 0.002	0.134 ± 0.026	0.076 ± 0.016	
Pituitary	0.042 ± 0.013	0.026 ± 0.008	0.173 ± 0.046	0.098 ± 0.026	0.035 ± 0.010	0.019 ± 0.006	0.147 ± 0.024	0.100 ± 0.019	
Salivary gland	0.145 ± 0.022	0.046 ± 0.010	1.332 ± 0.146	0.260 ± 0.044	0.120 ± 0.018	0.036 ± 0.008	0.869 ± 0.064	0.250 ± 0.030	
Thalamus	0.037 ± 0.010	0.014 ± 0.005	0.128 ± 0.038	0.053 ± 0.017	0.033 ± 0.011	0.011 ± 0.004	0.163 ± 0.066	0.067 ± 0.015	
Tongue	0.158 ± 0.026	0.074 ± 0.014	1.593 ± 0.167	0.609 ± 0.087	0.132 ± 0.021	0.061 ± 0.012	0.628 ± 0.053	0.319 ± 0.034	
Tooth	0.179 ± 0.017	0.086 ± 0.010	2.007 ± 0.141	0.592 ± 0.062	0.131 ± 0.014	0.071 ± 0.009	0.680 ± 0.035	0.330 ± 0.024	
Thyroid	0.196 ± 0.028	0.117 ± 0.018	41.380 ± 0.955	19.234 ± 0.541	0.158 ± 0.025	0.101 ± 0.017	34.340 ± 0.512	2.447 ± 0.094	
Heart	52.727 ± 0.592	6.888 ± 0.128	53.488 ± 0.732	8.521 ± 0.198	1.558 ± 0.085	0.322 ± 0.032	3.146 ± 0.154	0.701 ± 0.060	
Target breast	56.519 ± 0.617	52.100 ± 0.594	55.663 ± 0.757	51.275 ± 0.728	54.634 ± 0.586	51.258 ± 0.569	55.100 ± 0.592	51.101 ± 0.572	
Contralateral breast	0.737 ± 0.066	0.277 ± 0.038	1.135 ± 0.106	0.428 ± 0.061	0.673 ± 0.061	0.227 ± 0.032	1.504 ± 0.126	0.535 ± 0.068	
Ipsilateral lung	54.062 ± 0.666	9.789 ± 0.183	55.307 ± 1.242	13.386 ± 0.317	51.993 ± 0.633	6.704 ± 0.139	53.109 ± 0.661	11.692 ± 0.259	
Contralateral lung	0.724 ± 0.074	0.127 ± 0.027	1.400 ± 0.170	0.258 ± 0.054	0.493 ± 0.065	0.089 ± 0.021	0.979 ± 0.107	0.301 ± 0.057	
Diaphragm	38.051 ± 0.500	0.516 ± 0.034	46.609 ± 0.693	2.000 ± 0.074	47.251 ± 0.536	1.660 ± 0.047	51.629 ± 0.591	5.001 ± 0.126	
Trachea	0.494 ± 0.042	0.152 ± 0.020	35.650 ± 0.878	3.300 ± 0.169	0.501 ± 0.039	0.159 ± 0.019	10.319 ± 0.311	0.992 ± 0.062	
Esophagus	0.355 ± 0.033	0.201 ± 0.024	37.126 ± 0.903	6.183 ± 0.263	0.284 ± 0.031	0.157 ± 0.020	9.522 ± 0.239	1.278 ± 0.075	
Spinal cord	0.231 ± 0.026	0.056 ± 0.010	42.041 ± 0.965	0.776 ± 0.052	0.208 ± 0.024	0.046 ± 0.008	2.962 ± 0.162	0.323 ± 0.031	
Stomach	38.691 ± 0.503	0.498 ± 0.035	43.320 ± 0.661	1.387 ± 0.073	0.249 ± 0.026	0.085 ± 0.016	0.628 ± 0.060	0.258 ± 0.039	
Duodenum	0.136 ± 0.024	0.061 ± 0.013	0.273 ± 0.053	0.107 ± 0.023	0.119 ± 0.018	0.051 ± 0.010	0.397 ± 0.045	0.168 ± 0.027	
Small intestine	0.372 ± 0.033	0.043 ± 0.008	0.820 ± 0.073	0.076 ± 0.015	0.118 ± 0.023	0.020 ± 0.006	0.388 ± 0.071	0.069 ± 0.017	
Large intestine	10.962 ± 0.270	0.053 ± 0.008	43.602 ± 0.673	0.352 ± 0.020	0.162 ± 0.028	0.018 ± 0.005	0.466 ± 0.072	0.061 ± 0.015	
Liver	1.516 ± 0.084	0.130 ± 0.019	21.105 ± 0.474	0.268 ± 0.034	45.274 ± 0.523	0.438 ± 0.027	49.713 ± 0.570	2.156 ± 0.086	
Gall bladder	0.147 ± 0.030	0.071 ± 0.015	0.245 ± 0.047	0.119 ± 0.025	0.170 ± 0.019	0.084 ± 0.013	0.576 ± 0.061	0.267 ± 0.033	
Adrenal glands	0.171 ± 0.019	0.109 ± 0.015	0.327 ± 0.045	0.180 ± 0.027	0.112 ± 0.019	0.066 ± 0.012	0.352 ± 0.050	0.201 ± 0.030	
Rt kidney	0.096 ± 0.015	0.032 ± 0.008	0.164 ± 0.029	0.054 ± 0.015	0.111 ± 0.018	0.035 ± 0.008	0.304 ± 0.042	0.122 ± 0.022	
Lt kidney	0.181 ± 0.019	0.073 ± 0.012	0.296 ± 0.035	0.117 ± 0.020	0.092 ± 0.015	0.030 ± 0.008	0.401 ± 0.058	0.101 ± 0.021	
Pancreas	0.279 ± 0.028	0.147 ± 0.019	0.437 ± 0.047	0.242 ± 0.033	0.146 ± 0.028	0.065 ± 0.012	0.456 ± 0.060	0.205 ± 0.032	
Spleen	1.479 ± 0.085	0.229 ± 0.024	41.441 ± 0.634	0.466 ± 0.040	0.146 ± 0.019	0.047 ± 0.010	0.366 ± 0.044	0.155 ± 0.028	
Ovaries	0.016 ± 0.011	0.003 ± 0.002	0.030 ± 0.021	0.005 ± 0.003	0.018 ± 0.008	0.002 ± 0.001	0.051 ± 0.033	0.007 ± 0.004	
Uterus	0.018 ± 0.007	0.002 ± 0.002	0.037 ± 0.017	0.004 ± 0.003	0.016 ± 0.007	0.002 ± 0.001	0.060 ± 0.035	0.007 ± 0.004	
Vagina	0.009 ± 0.006	0.001 ± 0.001	0.020 ± 0.020	0.003 ± 0.002	0.008 ± 0.006	0.001 ± 0.001	0.042 ± 0.028	0.004 ± 0.002	
Bladder	0.018 ± 0.009	0.002 ± 0.001	0.031 ± 0.017	0.004 ± 0.002	0.012 ± 0.006	0.002 ± 0.001	0.065 ± 0.044	0.006 ± 0.003	
Cortical bone	49.505 ± 0.512	0.758 ± 0.013	57.124 ± 1.022	1.884 ± 0.039	48.748 ± 0.487	0.841 ± 0.012	51.832 ± 0.638	1.883 ± 0.032	
Bone marrow	53.734 ± 0.600	0.200 ± 0.006	60.303 ± 1.160	0.969 ± 0.024	52.367 ± 0.571	0.228 ± 0.006	56.829 ± 0.735	1.055 ± 0.021	
Cartilage	0.308 ± 0.030	0.053 ± 0.009	44.423 ± 0.983	1.256 ± 0.057	0.283 ± 0.028	0.045 ± 0.007	4.311 ± 0.188	0.228 ± 0.023	
Muscle	54.296 ± 0.602	1.411 ± 0.017	61.041 ± 1.169	3.362 ± 0.049	53.023 ± 0.572	0.869 ± 0.011	56.824 ± 0.739	2.409 ± 0.034	
Skin	49.770 ± 0.573	1.197 ± 0.016	49.502 ± 0.714	2.137 ± 0.038	46.633 ± 0.535	1.057 ± 0.014	55.069 ± 0.718	1.896 ± 0.035	

Comparison of out‐of‐field organ doses between WBRT alone and WBRT plus RNRT plans revealed that the latter was inclined to increase the maximum dose for out‐of‐field organs. Also, RNRT produced the mean dose increase in contrast to WBRT alone. The ratios of mean doses between WBRT alone and WBRT plus RNRT were approximately as large as 3.5–8.0 at the head and neck region and around 1.5–6.6 in the lower abdominal region. This increase resulted from scattering radiation which proceeded via regional lymph node fields. Table 3 summarizes EAR for out‐of‐field organs (per 10,000 PY at the age of 70 yr after exposure at the ages of 30 and 50 yr) for conventional radiotherapy techniques calculated using Eqs. (1) and (2). Additional RNRT increased to the same degree as that for EAR found for WBRT only. EAR of the stomach with WBRT plus RNRT for the left breast was 13.2, which was the highest value excluding the breast and lung. Other organs with EAR higher than 1 are the thyroid and esophagus which were reported to increase second cancer risk. Furthermore, the EARs of the large intestine and liver were also higher than 1. The EAR of other out‐of‐field organs was less than 1.

**Table 3:** EAR for out‐of‐field organs (per 10,000 PY at the age of 70 yr following exposure at the age of 30 and 50 yr) for WBRT for right and left breasts, and WBRT plus RNRT for right and left breasts, as calculated using dose–response models.

Site	Age at exposure							
	30				50			
	Left side irradiation		Right side irradiation		Left side irradiation		Right side irradiation	
	WBRT	WBRT plus RNRT	WBRT	WBRT plus RNRT	WBRT	WBRT plus RNRT	WBRT	WBRT plus RNRT
Ipsilateral lung	14.0	20.5	11.6	17.6	14.6	21.3	12.1	18.3
	(9.51; 18.7)	(14.0; 27.4)	(7.89; 15.5)	(11.9; 23.4)	(8.58; 22.4)	(12.6; 32.8)	(7.12; 18.6)	(10.8; 28.1)
\Stomach	4.73	13.2	0.810	2.45	4.58	12.8	0.785	2.38
	(3.04; 6.97)	(8.46; 19.4)	(0.520; 1.19)	(1.58; 3.62)	(2.09; 7.96)	(5.83; 22.2)	(0.358; 1.36)	(1.08; 4.13)
Thyroid	0.141	5.50	0.121	1.38	0.0469	1.83	0.0405	0.460
	(0.0586; 0.258)	(2.29; 10.1)	(0.0506; 0.223)	(0.575; 2.53)	(0.00; 0.152)	(0.00; 5.96)	(0.00; 0.132)	(0; 1.50)
Contralateral breast	2.55	3.94	2.08	4.93	1.02	1.58	0.838	1.98
	(1.88; 3.32)	(2.91; 5.14)	(1.54; 2.72)	(3.64; 6.42)	(0.582; 1.63)	(0.899; 2.53)	(0.476; 1.34)	(1.12; 3.16)
Large intestine	0.424	2.81	0.147	0.492	0.0847	0.562	0.0293	0.0983
	(0.233; 0.636)	(1.55; 4.22)	(0.0807; 0.22)	(0.27; 0.737)	(0.0159; 0.207)	(0.105; 1.37)	(0.0055; 0.0715)	(0.0184; 0.240)
Contralateral lung	0.954	1.94	0.665	2.26	0.992	2.02	0.692	2.35
	(0.649; 1.27)	(1.32; 2.58)	(0.452; 0.887)	(1.53; 3.01)	(0.585; 1.53)	(1.19; 3.10)	(0.408; 1.06)	(1.38; 3.61)
Esophagus	0.116	1.52	0.0912	0.741				
	(0.0361; 0.221)	(0.47; 2.87)	(0.0283; 0.173)	(0.23; 1.41)				
Liver	0.560	1.15	1.88	1.45	0.339	0.696	1.14	0.879
	(0.00; 0.938)	(0.00; 1.93)	(0.00; 3.15)	(0.00; 2.43)	(0.0652; 0.834)	(0.134; 1.71)	(0.219; 2.80)	(0.169; 2.16)
Tongue	0.0415	0.341	0.0340	0.178				
	(0.0148; 0.0889)	(0.122; 0.731)	(0.0121; 0.0729)	(0.0637; 0.382)				
Skin	0.419	0.119	0.370	0.664	0.436	0.123	0.385	0.690
	(0.0359; 1.32)	(0.0102; 0.373)	(0.0317; 1.16)	(0.0569; 2.09)	(0.00; 0.347)	(0.00; 0.0983)	(0.00; 0.307)	(0.00; 0.550)
Pancreas	0.0676	0.111	0.0298	0.0944				
	(0.00; 0.221)	(0.00; 0.362)	(0.00; 0.0971)	(0.00; 0.308)				
Pituitary	0.0132	0.0502	0.0099	0.051				
	(0.0044; 0.0246)	(0.0167; 0.0934)	(0.0033; 0.0184)	(0.017; 0.0948)				
Cerebellum	0.0090	0.0394	0.0074	0.0574				
	(0.0030; 0.0167)	(0.0131; 0.0735)	(0.0025; 0.0138)	(0.0191; 0.107)				
Hypothalamus	0.0096	0.0376	0.0065	0.0424				
	(0.0032; 0.0178)	(0.0125; 0.07)	(0.0022; 0.012)	(0.0141; 0.079)				
Thalamus	0.0069	0.0272	0.0057	0.0343				
	(0.0023; 0.0129)	(0.0091; 0.0506)	(0.0019; 0.0105)	(0.0114; 0.0638)				
Gray matter	0.0067	0.0259	0.0058	0.0333				
	(0.0022; 0.0125)	(0.0086; 0.0482)	(0.0019; 0.0108)	(0.0111; 0.062)				
White matter	0.0067	0.0256	0.0057	0.033				
	(0.0022; 0.0124)	(0.0085; 0.0476)	(0.0019; 0.0106)	(0.011; 0.0613)				
Bladder	0.0073	0.0131	0.0051	0.0199	0.0048	0.0086	0.0034	0.0131
	(0.0025; 0.0123)	(0.0045; 0.0221)	(0.0018; 0.0087)	(0.0069; 0.0337)	(0.0011; 0.0102)	(0.0021; 0.0184)	(0.0008; 0.0072)	(0.0031; 0.0280)
Lt kidney	0.0058	0.0093	0.0024	0.0081				
	(0.00; 0.0319)	(0.00; 0.0513)	(0.00; 0.0133)	(0.00; 0.0445)				
Rt kidney	0.0026	0.0043	0.0028	0.0098				
	(0.00; 0.0142)	(0.00; 0.0237)	(0.00; 0.0154)	(0.00; 0.0538)				
Ovary	0.0016	0.0029	0.0011	0.0041				
	(0.0001; 0.0037)	(0.0001; 0.0067)	(0.0000; 0.0026)	(0.0002; 0.0096)				
Uterus	0.0014	0.0025	0.0010	0.0039				
	(0.00; 0.0047)	(0.00; 0.0083)	(0.00; 0.0034)	(0.00; 0.0132)				
Gall bladder	0.00	0.00	0.00	0.00				
	(0.00; 0.0362)	(0.00; 0.0608)	(0.00; 0.0427)	(0.00; 0.136)				

The linear‐exponential dose–response model was used for organs exposed over 2 Gy. The linear dose–response model was used for other organs. EAR based on Japanese atomic bomb survivor data is not reported if no value is shown. Values in parentheses are 90% confidence intervals.

## 4 Discussion

This study simulated absorbed doses for 43 organs and estimated the risk of second cancer for major organs attributed to specific internal body scatter from breast radiotherapy with WBRT alone and WBRT plus RNRT using full MC code PHITS in the complete whole‐body female model. The simulated mean absorbed dose for the target breast matched with TPS within 1%. Substantial differences were found, however, between them for the low‐dose bath such as the lung and heart (Fig. 4). In that regard, the estimated uterus and ovary doses were consistent with reported doses because of internal body scatter at the fetal level from breast radiotherapy with two opposing tangential fields.^14^

Comparison of the out‐of‐field organ doses between those found for WBRT alone and WBRT plus RNRT indicates that the latter greatly increased the absorbed doses for most out‐of‐field organs. As one might expect, the exposure dose for organs around the head and neck increases because of opposing regional lymph node fields in RNRT. Furthermore, these fields markedly increased the dose for all out‐of‐field organs including the lower abdomen. Although these additional fields did not overlap with opposing tangential fields for the breast, the scattered radiation to out‐of‐field organs originated from each field was not negligible.

Modern photon radiotherapy techniques such as intensity‐modulated radiation therapy (IMRT) and volumetric modulated arc therapy (VMAT) have also become the standard of care for multiple cancer sites. They involve a higher number of beams than conventional radiotherapy techniques. Therefore, the dose can be concentrated into a target region. Such techniques might increase the low‐dose bath region.^38^, ^39^, ^40^ The influences of IMRT and VMAT upon deterministic effects and second cancer risk represent a controversial issue.^12^, ^13^, ^41^, ^42^, ^43^ The MC dose simulation on the whole‐body female model can also be expected to help to evaluate acquired risk factors of modern photon radiotherapy.

The convolution–superposition algorithm in TPS overestimated the dose for peripheral organs. For example, many reports have described dose overestimations around the lung caused by the scatter kernel of the convolution–superposition algorithm.^35^, ^36^, ^37^ The difference between PHITS and TPS might have been caused by the treatment of secondary electron components in low‐density inhomogeneous media. The lateral electronic equilibrium is difficult to model because the range of secondary electrons in low‐density inhomogeneous media, such as the air and lung, is longer than that in water. By contrast, MC simulation in this study was conducted with actual elemental compositions that are allocated directly to each tissue on the whole‐body model. Therefore, the MC simulation algorithm is expected to be valid for estimation of a low‐dose bath including regions that are distant from the target.

The contribution of internal body scatter over lead leakage has been reported in the literature.^14^ Nevertheless, no report of the literature presents data for specific organ doses and second cancer risks posed by internal body scatter in breast radiotherapy. Our simulation results demonstrate clearly that unavoidable EAR for out‐of‐field organs was less than 1. This result indicates that the risk of second cancer is much lower than for the breast, lung, thyroid, and esophagus which were earlier reported to show increased risk. The risk might be negligible for these organs. However, the skin on the surface of the human body might be more affected by head leakage, collimator transmission, and room scattering from all directions than the internal body organs. Evaluating the second cancer risk for the skin requires further investigation. Although the risk is small, WBRT plus RNRT elevates the risk of the second cancer of out‐of‐field organs considerably, by as much as 3.5–8 times for the head and neck region, compared to WBRT according to the dose calculation conducted for this study. That risk is nonetheless acceptable because radiotherapy benefits outweigh the attendant risk.

Medical safety demands our best efforts to reduce this risk. The relation between radiation therapy and second cancer is being clarified.^3^, ^44^, ^45^ Combining the specific organ doses obtained in this study with prognosis of the patient is expected to clarify the causal relation between the absorbed dose and second cancer in breast radiotherapy.

Regarding the effect of uncertainty in dose estimation on estimating the risk of second cancer, reports of the relevant literature claim that 50% uncertainty in the calculated peripheral dose is acceptable given the uncertainties of risk factors for second cancers.^46^ However, the risk of second cancer might be different when a dose uncertainty of at least 50% exists.^47^ In our study, the statistical uncertainties of out‐of‐field organ doses were mostly less than 50%. Therefore, assuming 50% as the threshold of dose uncertainty for risk estimation based on the former claim, then the statistical uncertainties of organ doses which have less than 50% uncertainty can be acceptable for risk estimation in our study. However, the uncertainties for some organs such as the bladder, uterus, and ovaries exceeded 50%. The estimated risks of second cancer for these organs might be inaccurate based on the assumption, but the risks would still be sufficiently small as to be negligible.

Limitations exist in this study. First, only one whole‐body female model was used for dose calculations. Reportedly, the patient’s body type has little effect on a low‐dose bath.^48^ However, DVHs for peripheral organs differ considerably among patients. Treatment plans such as different irradiation of right and left breasts were examined in this study. Therefore, when estimating the risk of second cancer for peripheral organs, they are calculable with the patient's individual CT dataset used during treatment planning.

A second limitation is the coverage of Japanese atomic bomb survivor data. Marked differences exist in the rates of some cancers, such as breast and ovary cancer, between the general Japanese population and the world population.^49^ This striking interpopulation difference in baseline rates for some cancers presents a complicated issue, as discussed in earlier reports.^50^, ^51^ Moreover, the EAR data include no distinction between men and women. Therefore, it is difficult to apply the data to the rates of some cancers for which gender differences are large, such as bladder cancer. Extrapolating the results to the world population is hindered by these inherent limitations.

## 5 Conclusion

We applied MC dose simulation and estimated the second cancer risks for a low‐dose bath by internal body scatter for WBRT alone and WBRT plus RNRT with realistic radiotherapy conditions using a postured whole‐body female model. The MC simulations based on sophisticated physics can validate low‐dose bath attributable mainly to internal body scatter. Most out‐of‐field organs present sufficiently low second cancer risk. The data suggest that conventional breast radiotherapy contributes little to the probability of second cancer of the breast with shielding head scatter. Further MC dose calculation on clinical patient data is expected to help in evaluating acquired risk factors for patients considering or undergoing radiotherapy.

## Conflict of Interest

The authors have no relevant affiliations or financial interests to report.
